# ICGA-Guided Decision-Making Is Associated with Lower Flap Necrosis Rates in Pre-Expanded Extended Lower Trapezius Myocutaneous Flaps

**DOI:** 10.3390/jcm15166262

**Published:** 2026-08-13

**Authors:** Miao Wang, Yuanbo Liu

**Affiliations:** Department of Plastic and Reconstructive Surgery, Plastic Surgery Hospital, Chinese Academy of Medical Sciences and Peking Union Medical College, No. 33 Ba-Da-Chu Road, Beijing 100144, China; drdamienw@pumc.edu.cn

**Keywords:** indocyanine green angiography, extended lower trapezius myocutaneous flap, flap necrosis, intraoperative decision-making, flap perfusion

## Abstract

**Background:** Evidence regarding the association between indocyanine green angiography (ICGA) use and flap necrosis remains inconsistent. This inconsistency may partly reflect differences in how studies define comparison groups, particularly whether classification is based on ICGA use or on whether ICGA findings informed the final intraoperative decision. We therefore compared flap necrosis primarily according to the basis for the final decision and secondarily according to ICGA use. **Methods:** This single-center retrospective comparative cohort study included 108 pre-expanded extended lower trapezius myocutaneous (e-LTMC) flaps in 101 patients between 2012 and 2022. Flaps were classified into the clinical-assessment group (Group A, *n* = 49) and the ICGA-guided group (Group B, *n* = 59) according to the basis for the final decision. Outcomes were overall and severe flap necrosis. Firth penalized logistic regression was the primary inferential method. **Results:** Overall necrosis occurred in 4/59 flaps (6.8%) in Group B and 14/49 (28.6%) in Group A (adjusted odds ratio [OR] 0.223, 95% confidence interval [CI] 0.063–0.661, *p* = 0.006). Severe flap necrosis occurred in 1/59 flaps (1.7%) and 11/49 (22.4%), respectively (adjusted OR 0.097, 95% CI 0.010–0.436, *p* = 0.001). Comparisons based on ICGA use alone showed no clear difference in either outcome (*p* = 0.422 and *p* = 0.750). Excluding the 11 retained hypoperfused flaps attenuated the difference in overall necrosis (*p* = 0.056), whereas the difference in severe necrosis remained significant (*p* = 0.033). **Conclusions:** In pre-expanded e-LTMC flap reconstruction, a management pathway in which ICGA findings informed or confirmed the final intraoperative decision was associated with lower observed rates and lower adjusted odds of overall and severe flap necrosis. The observed association reflects the management pathway, including perfusion status and the feasibility of modification, rather than ICGA alone. ICGA is therefore best incorporated into intraoperative decision-making alongside clinical assessment.

## 1. Introduction

An accurate assessment of tissue perfusion is essential for success in any reconstructive scenario [1]. Tissue necrosis may compromise the reconstruction outcome with devastating effects on patients [2], such as prolonged hospitalization, additional revision procedures, increased healthcare costs, and potential delays in subsequent treatment [3,4]. Conventional clinical assessment remains the standard intraoperative approach to flap perfusion assessment and includes a subjective evaluation of skin color, temperature, capillary refill, and dermal edge bleeding. However, clinical assessment may not reliably identify tissue hypoperfusion [5]. These limitations have encouraged the use of adjunctive perfusion-assessment technologies. Indocyanine green angiography (ICGA) is a laser-assisted near-infrared fluorescence technique that allows real-time intraoperative visualization of flap perfusion. Still et al. [6] first evaluated flap perfusion with ICGA in 1999, and ICGA has since been applied across multiple reconstructive settings [3,4,7,8,9,10].

Whether ICGA use is associated with lower rates of flap necrosis remains uncertain. Some studies reported lower postoperative flap necrosis or complication rates with ICGA use [3,4,7], whereas others reported no clear difference [8,9]. A recent systematic review and meta-analysis of 25 studies reported an association between ICGA use and a lower risk of total flap loss in head and neck reconstruction but showed inconsistent associations across reconstructive settings [10]. As summarized in Table S1, three sources of variation recur across this literature [7,8,10,11,12]. First, flap physiology and reconstructive setting differ substantially between studies, so associations observed in one flap type or anatomical region may not readily apply to another [7,10]. Second, some comparative studies left it unclear whether ICGA findings changed or merely confirmed final intraoperative management [8,12]. Third, outcome definitions and reported endpoints are heterogeneous. Definitions of necrosis vary according to lesion size, timing of assessment, and mode of detection [11]. Reported endpoints also differ. Some studies used composite surgical-site or wound-related outcomes [13], others applied study-specific necrosis criteria [14,15], and others reported broader complication endpoints that combined necrosis with other postoperative events [16,17,18]. The second source of variation is particularly relevant to the present study. Whether ICGA was performed is not equivalent to whether ICGA findings informed the final intraoperative decision. Studies defining comparison groups in these two ways therefore address related but distinct clinical questions.

The lower trapezius myocutaneous flap is an established option for head and neck reconstruction [19,20,21,22,23]. Rosen [24] first described the extended lower trapezius myocutaneous (e-LTMC) flap, in which the skin island extends distally beyond the inferior border of the trapezius muscle to facilitate coverage of remote defects. Perfusion of this distal portion may therefore be less predictable [24,25,26]. Pre-expansion can provide a larger and thinner skin island, facilitating coverage of extensive or remote defects [22,23]. Postoperative flap necrosis rates in e-LTMC flaps have been reported to be as high as 44%, with necrosis typically involving the distal portion [24,25]. Transfer, rotation, or passage through a subcutaneous tunnel may further alter distal perfusion, while coverage requirements constrain how much potentially compromised tissue can be removed [22]. The clinical challenge is therefore not only to identify distal hypoperfusion but also to determine whether and how the flap can be modified without compromising adequate defect coverage.

Building on our previous report of ICGA-assessed pre-expanded e-LTMC flaps [22], the present study examined a partially overlapping cohort that also included flaps managed without ICGA. It therefore represents an expanded exploratory analysis addressing related but distinct questions rather than an independent validation of the earlier report. The primary objective was to evaluate the association between the basis for final intraoperative decision-making and overall and severe flap necrosis. The secondary objective was to compare the same outcomes according to ICGA use.

## 2. Materials and Methods

### 2.1. Study Design and Cohort Selection

This retrospective comparative cohort study reviewed the records of all consecutive lower trapezius myocutaneous flap reconstructions performed at a single center between 2012 and 2022. The study was approved by the Ethics Committee of Plastic Surgery Hospital, Chinese Academy of Medical Sciences and Peking Union Medical College (approval Nos. 2020-200, 2022-142, and 2026-234). A total of 153 flaps were screened. Twenty-one flaps were excluded because they were not pre-expanded, 19 because the procedures involved collaboration with other centers and therefore did not meet the single-center eligibility criterion, and five because the operative or follow-up records were incomplete. The final cohort therefore comprised 108 pre-expanded e-LTMC flaps in 101 patients. All included procedures were performed by the same senior surgeon, and the flap was the unit of analysis. Because cases with incomplete records were excluded during cohort selection, no missing data were present in the analyzed variables. Retrieved parameters included demographic features, preoperative comorbidities, defect characteristics, expander size, expansion time, flap dimensions, and intraoperative management. The study conformed to the ethical principles of the Declaration of Helsinki.

Flaps were assigned to Group A (clinical-assessment group) or Group B (ICGA-guided group) according to the basis for final intraoperative decision-making. For the present analysis, this analytical classification was applied retrospectively during case-level data review rather than prospectively at the time of surgery. Group classification was based on the contemporaneous operative records, the actual intraoperative management performed, and, where available, the systematically re-reviewed ICGA recordings, using the analytical criteria described below. Group A included flaps in which ICGA was not performed and management was based on clinical assessment alone, together with flaps in which ICGA identified distal hypoperfusion, but the flap was retained without modification because further excision would have compromised adequate defect coverage and concurrent clinical assessment revealed no overt ischemia. Group B included flaps in which final intraoperative management followed the ICGA findings, including flaps with ICGA-confirmed adequate perfusion, flaps that underwent ICGA-guided prophylactic excision, and flaps in which the hypoperfused distal portion was trimmed for use as a full-thickness skin graft. Group allocation was therefore jointly influenced by the intraoperative perfusion status, the feasibility of removing hypoperfused tissue while maintaining adequate defect coverage, and the modification actually performed. These components could not be separated within the present retrospective design.

Of the 108 flaps included in the present study, 69 had been included in our previous report [22], which described ICGA findings, intraoperative management strategies, and necrosis outcomes within an ICGA-assessed cohort. The present study included 39 additional flaps, comprising 38 flaps in which ICGA was not performed and one additional ICGA-assessed flap, and evaluated a different research question by examining both ICGA use and the basis for final intraoperative decision-making in the expanded cohort. The present study should therefore be regarded as an expanded exploratory analysis of a partially overlapping cohort rather than an independent validation of the previous findings.

### 2.2. Outcomes

The study outcomes were overall flap necrosis and severe flap necrosis. Postoperative flap status was determined retrospectively from serial clinical assessments documented during hospitalization and follow-up. Flap status was recorded on postoperative days 7 and 14, and the status on postoperative day 14 was used to determine whether necrosis had occurred. Overall flap necrosis was defined as any clinically evident partial or total loss of flap tissue. Necrosis was classified as mild or severe according to the postoperative course and overall treatment burden, incorporating follow-up information beyond postoperative day 14. Mild necrosis was limited and resolved with routine postoperative care or conservative dressing changes. Severe necrosis represented clinically significant tissue loss requiring active wound management beyond routine postoperative care, including repeated outpatient wound care beyond routine dressing changes, in-office or operative debridement, skin grafting, or additional flap transfer. This clinical severity classification differs from the anatomical partial- versus full-thickness classification used in our previous report [22], and the two grading systems are not directly interchangeable.

### 2.3. Procedures

**Figure 1:**
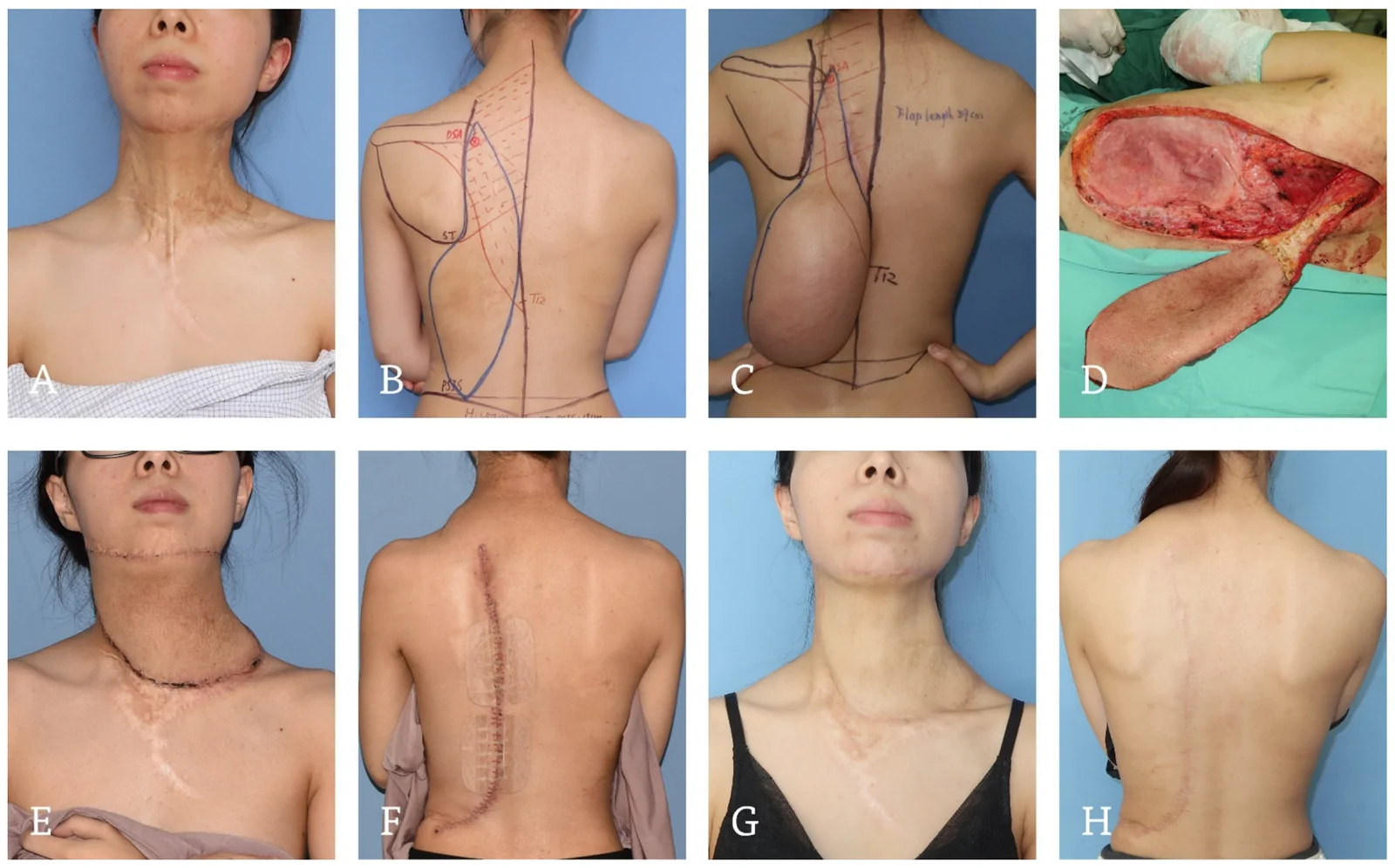
Representative case of two-stage reconstruction using a pre-expanded extended lower trapezius myocutaneous flap. (**A**) Preoperative appearance of the cervical scar contracture. (**B**) Flap and expander design before the first-stage procedure, with the anticipated course of the dorsal scapular artery marked. (**C**) Expanded donor site and flap design before the second-stage reconstruction. (**D**) Elevation of the pre-expanded extended lower trapezius myocutaneous flap. (**E**) Appearance of the recipient site on postoperative day 14. (**F**) Appearance of the donor site on postoperative day 14. (**G**) Follow-up appearance of the reconstructed cervical region approximately 14 months after the initial reconstruction and after a secondary flap-thinning procedure. (**H**) Follow-up appearance of the donor site at the same time point. DSA, dorsal scapular artery; e-LTMC, extended lower trapezius myocutaneous flap.

All reconstructions were performed by the same senior surgeon using a two-stage procedure. In the first stage, a tissue expander (Shanghai Weining Plastic Products Co., Ltd., Shanghai, China) was placed in the sub-trapezius plane and progressively expanded until sufficient tissue was obtained for reconstruction. In the second stage, the pre-expanded e-LTMC flap was designed along the course of the dorsal scapular artery, elevated with the expanded skin paddle and lower trapezius muscle, and transferred to the recipient site through a subcutaneous tunnel or as a propeller flap according to the location and geometry of the defect. A representative case illustrating the two-stage reconstructive procedure is shown in Figure 1.

Among flaps assessed with ICGA, the same standardized protocol was applied at two time points using the SPY Elite system (LifeCell Corp., Branchburg, NJ, USA). The initial examination followed flap harvest and pedicle dissection. The second examination followed flap transfer and inset at the recipient site. In Group B, findings from the second examination served as the basis for final intraoperative decision-making. During each examination, the operating room was darkened and all surgical lights were extinguished. A fixed distance of 30 cm was maintained between the flap and the imaging head. Indocyanine green at a weight-based dose of 0.2 mg/kg (Dandong Yichuang Pharmaceutical Co., Ltd., Dandong, China) was intravenously administered, followed by a 10 mL flush with normal saline. The SPY Elite system recorded fluorescence flow and displayed real-time grayscale images at 7.5 frames/s for at least 136 s after ICG administration. Observation continued for a further 136 s in selected cases. The first and subsequent 136 s segments of a representative continuous recording are provided in Video S1 and Video S2, respectively. Fluorescence patterns were assessed qualitatively by subjective visual interpretation rather than quantitative fluorescence analysis. Well-perfused areas appeared bright, whereas hypoperfused areas appeared relatively dark [27]. In selected cases, an additional ICGA examination was performed after flap modification to confirm adequate perfusion of the remaining flap. No predefined quantitative fluorescence threshold was applied. For the present study, all intraoperative ICGA recordings were systematically re-reviewed using uniform qualitative criteria based on the complete fluorescence sequence to standardize the perfusion classification used for analysis. Areas showing absent or markedly delayed fluorescence relative to the proximal portion of the flap were classified as hypoperfused. The operative intervention performed in each case was determined from the contemporaneous operative records.

In Group A, surgeons identified areas of suspected hypoperfusion by conventional clinical assessment. Clinical criteria included pale or blue-purple skin color, slow capillary refill, and dark bleeding at the skin edges. In Group B, the senior surgeon delineated the boundaries of hypoperfused areas in real time by visual assessment of the ICGA fluorescence sequence. For areas selected for intervention in either group, surgeons performed prophylactic excision when adequate defect coverage could be maintained. When excision would compromise adequate defect coverage, surgeons trimmed the hypoperfused distal portion for use as a full-thickness skin graft.

The flap was inset, and the donor site was closed primarily. Patients were placed in the lateral decubitus position for the first three postoperative days to avoid external pedicle compression. Skin color, capillary refill, and skin temperature were assessed hourly for the first 24 h and every two hours for the subsequent 48 h. Sutures were removed 14 days postoperatively. Patients with flap necrosis were managed on a case-by-case basis with conservative dressing changes, split-thickness skin grafting, or secondary flap transfer. All patients were followed up for at least six months after surgery.

### 2.4. Statistical Analysis

Categorical variables were summarized as frequencies and percentages, and continuous variables as means with standard deviations. Continuous variables were compared between groups using Welch’s *t* test, whereas categorical variables and event rates were compared using Fisher’s exact test.

Because only 18 overall flap necrosis events and 12 severe flap necrosis events occurred, Firth penalized logistic regression was used as the primary inferential method to reduce small-sample and separation bias. Odds ratios (ORs) were reported with 95% confidence intervals (CIs) derived from the profile penalized likelihood. For crude group comparisons, including the sensitivity analyses, *p* values were obtained using Fisher’s exact test. For Firth regression models, *p* values were obtained using penalized likelihood ratio tests. Covariates were selected on clinical grounds rather than by univariable *p*-value screening. Separate univariable Firth penalized logistic regression models were fitted for each candidate variable for descriptive purposes. These analyses were not used to select covariates for the multivariable models. For each outcome, an unadjusted model was followed by a model adjusted for transfer method and an extended model additionally adjusted for defect area. Model complexity was limited according to the number of outcome events.

The main analysis compared the ICGA-guided group with the clinical-assessment group. A secondary analysis compared flaps in which ICGA was and was not performed. Sensitivity analyses excluded flaps with ICGA-detected hypoperfusion that were retained without modification, restricted the cohort to procedures performed from 2016 onward, and applied both restrictions simultaneously. Among flaps with ICGA-detected distal hypoperfusion, the comparison between modified and retained flaps was conducted as a post hoc exploratory subgroup analysis.

Analyses were performed using R version 4.3.3 (R Foundation for Statistical Computing, Vienna, Austria) and the logistf package, version 1.26.0. All tests were two-sided, and *p* < 0.05 was considered statistically significant.

## 3. Results

### 3.1. Cohort Characteristics

**Figure 2:**
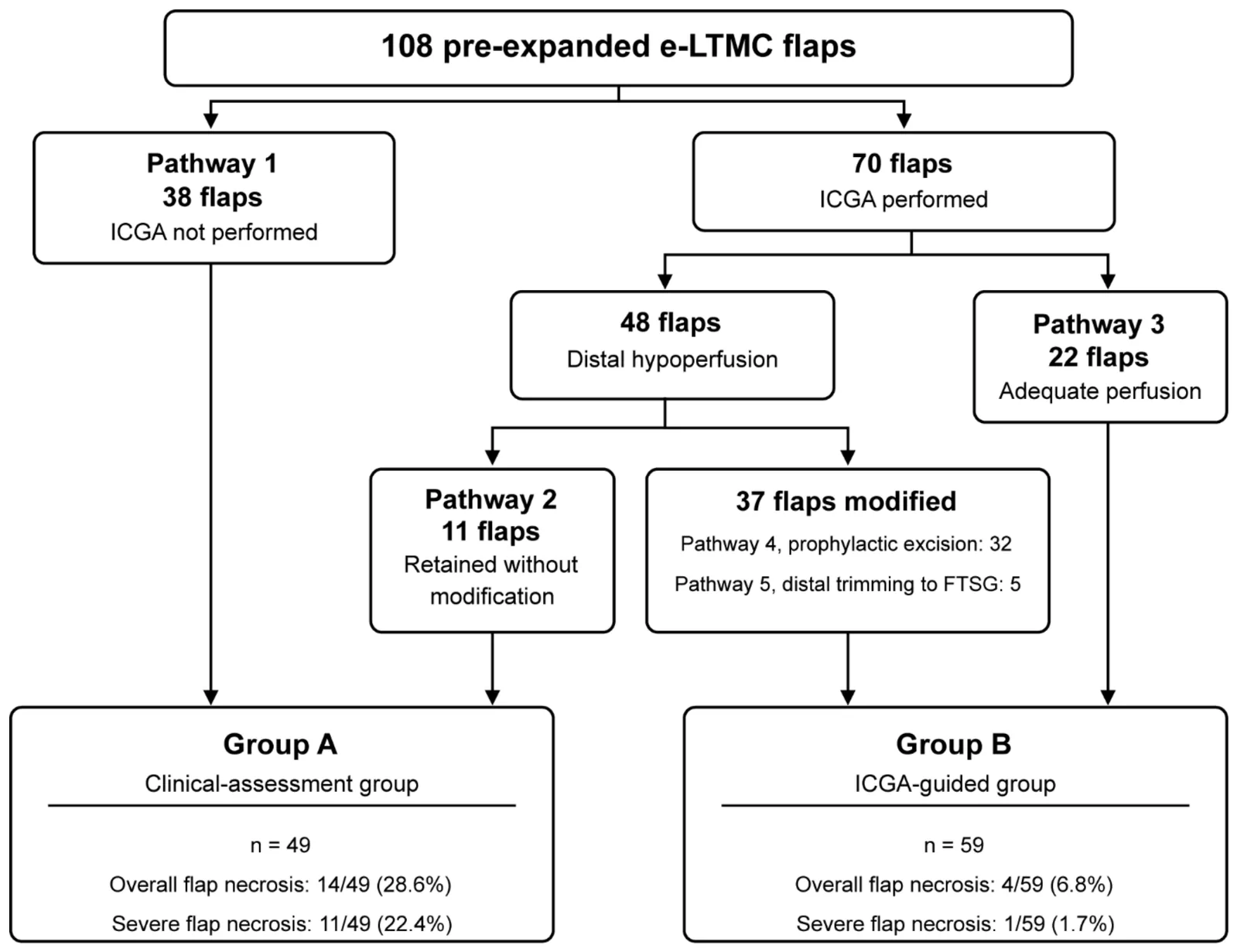
Cohort flow, intraoperative decision pathways, derivation of the analytical groups, and postoperative flap-necrosis outcomes. Of the 108 pre-expanded e-LTMC flaps, ICGA was not performed in 38 flaps (pathway 1). Among the 70 flaps assessed with ICGA, 48 showed distal hypoperfusion and 22 were classified as having adequate perfusion on systematic re-review (pathway 3). Of the 48 flaps with distal hypoperfusion, 11 were retained without modification (pathway 2), 32 underwent prophylactic excision (pathway 4), and 5 underwent distal trimming for use as a full-thickness skin graft (pathway 5). Group A comprised pathways 1 and 2, whereas Group B comprised pathways 3–5. e-LTMC, extended lower trapezius myocutaneous; FTSG, full-thickness skin graft; ICGA, indocyanine green angiography.

Of 153 lower trapezius myocutaneous flaps screened, 108 pre-expanded e-LTMC flaps in 101 patients were included in the final cohort after exclusion of 21 non-pre-expanded flaps, 19 multicenter collaborative cases, and five flaps with incomplete records. Of the 108 flap procedures, 61 involved male patients and 47 involved female patients. The mean age was 16.0 ± 11.0 years, and the mean body mass index was 19.8 ± 3.6 kg/m^2^. Most defects resulted from post-burn scar contracture (98/108, 90.7%) and involved the neck (96/108, 88.9%). Baseline demographic, defect, expansion, flap, and transfer characteristics are presented in Table 1. Group B had greater defect width (12.4 ± 2.2 cm vs. 11.0 ± 2.5 cm; *p* = 0.003), longer expansion duration (29.8 ± 9.7 weeks vs. 24.8 ± 6.5 weeks; *p* = 0.002), and a higher frequency of an unexpanded distal component (31/59, 52.5% vs. 11/49, 22.4%; *p* = 0.002). All donor sites were closed primarily. No adverse effects related to ICG administration were documented in the ICGA-assessed subgroup. No total flap necrosis occurred. Overall flap necrosis, which in all cases affected the distal portion of the flap, occurred in 18/108 flaps (16.7%), including mild necrosis in 6/108 flaps (5.6%) and severe necrosis in 12/108 flaps (11.1%). The classification of all 108 flaps according to the basis for final intraoperative decision-making and the corresponding postoperative flap necrosis outcomes are summarized in Figure 2 and Table 2.

### 3.2. Group-Specific Intraoperative Management and Flap Outcomes

Group A comprised 49 flaps: 38 were managed without ICGA, and 11 had ICGA-detected distal hypoperfusion but were retained without modification. Among the 38 flaps managed without ICGA, 35 were retained without modification, one underwent excision, and two underwent distal trimming. Overall flap necrosis occurred in 14/49 flaps (28.6%), including mild necrosis in 3 flaps and severe flap necrosis in 11 flaps.

Group B comprised 59 flaps. Overall flap necrosis occurred in 4/59 flaps (6.8%), including mild necrosis in 3 flaps and severe flap necrosis in 1 flap.

**Figure 3:**
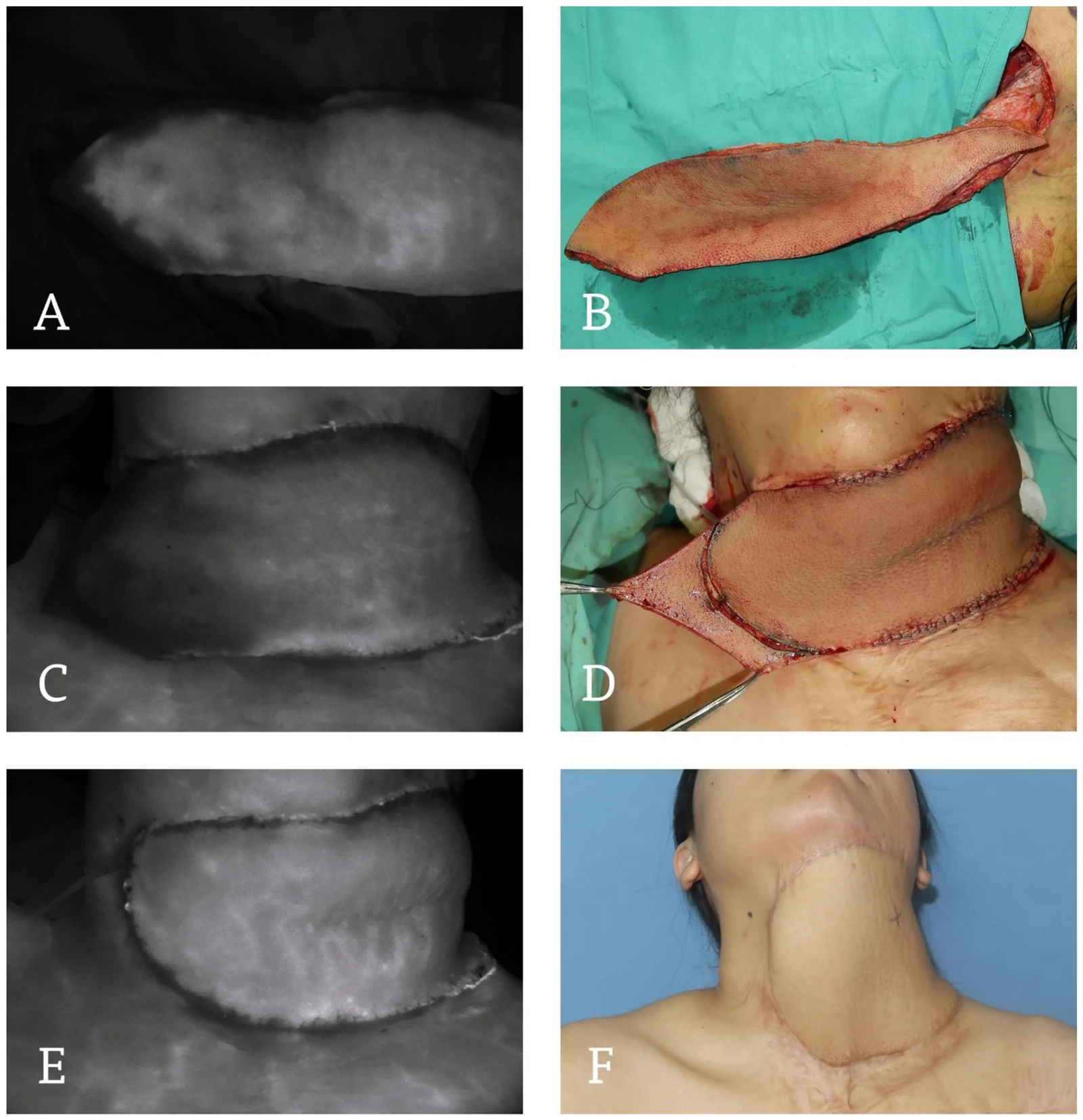
Representative case in the ICGA-guided group (pathway 4). (**A**) ICGA performed after flap harvest and pedicle dissection demonstrated the initial perfusion pattern of the flap. (**B**) Clinical photograph of the harvested flap before transfer. (**C**) After flap transfer to the recipient site, repeat ICGA identified a hypoperfused area at the distal portion of the flap. (**D**) The hypoperfused distal portion was excised on the basis of the ICGA findings. (**E**) Additional ICGA after excision showed adequate perfusion of the remaining flap. (**F**) Follow-up photograph at six months after surgery showed complete wound healing without flap necrosis. ICGA, indocyanine green angiography.

**Figure 4:**
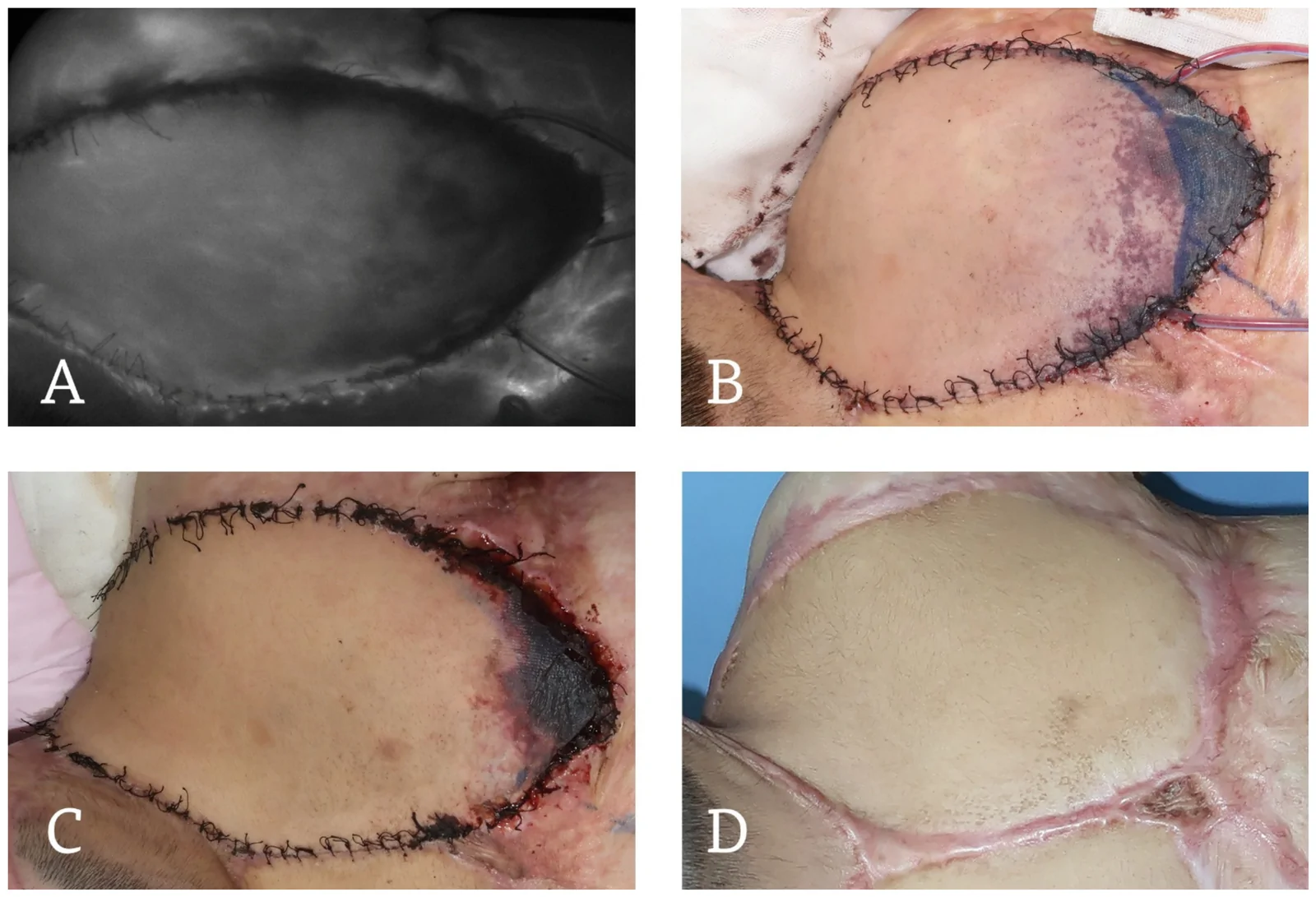
Representative case in the clinical-assessment group (pathway 2) in which ICGA identified distal hypoperfusion. The flap was retained without modification because further excision would compromise adequate defect coverage and concurrent clinical assessment revealed no overt ischemia. (**A**) After flap transfer and inset, ICGA identified hypoperfusion at the distal portion of the flap. (**B**) Distal flap ischemia was evident on postoperative day four. (**C**) Distal flap necrosis was evident two weeks after surgery. (**D**) Follow-up photograph at eight months after surgery showed a healed wound. ICGA, indocyanine green angiography.

ICGA was performed in 70 flaps. On systematic re-review, 22 flaps were classified as having adequate perfusion and 48 as having distal hypoperfusion. Of the 48 hypoperfused flaps, 32 underwent prophylactic excision, five had the hypoperfused distal portion trimmed for use as a full-thickness skin graft, and 11 were retained without modification. Representative cases of prophylactic excision and retention without modification are shown in Figure 3 and Figure 4, respectively. The intraoperative pathways and corresponding necrosis outcomes are summarized in Table 2.

### 3.3. Comparison of Flap Necrosis Rates

In the primary comparison, overall flap necrosis occurred in 4/59 flaps (6.8%) in Group B and in 14/49 flaps (28.6%) in Group A (Fisher’s exact test, *p* = 0.004; Firth OR, 0.199; 95% CI, 0.057–0.581). Severe flap necrosis occurred in 1/59 flaps (1.7%) and in 11/49 flaps (22.4%), respectively (Fisher’s exact test, *p* = 0.001; Firth OR, 0.086; 95% CI, 0.009–0.383) (Table 3).

In the secondary analysis based on whether ICGA was performed, overall flap necrosis occurred in 10/70 flaps (14.3%) in which ICGA was performed and in 8/38 flaps (21.1%) in which ICGA was not performed (Fisher’s exact test, *p* = 0.422; Firth OR, 0.623; 95% CI, 0.228–1.737). Severe flap necrosis occurred in 7/70 flaps (10.0%) and in 5/38 flaps (13.2%), respectively (Fisher’s exact test, *p* = 0.750; Firth OR, 0.719; 95% CI, 0.222–2.456) (Table 3).

In a post hoc exploratory analysis restricted to the 48 flaps with ICGA-detected distal hypoperfusion, 37 flaps were modified by prophylactic excision or distal trimming for use as a full-thickness skin graft, whereas 11 were retained without modification. Baseline and operative characteristics of the two subgroups are presented in Table S2. Overall flap necrosis occurred in 3/37 modified flaps (8.1%) and in 6/11 retained flaps (54.5%) (Fisher’s exact test, *p* = 0.002; Firth OR, 0.086; 95% CI, 0.016–0.393). Severe flap necrosis occurred in 1/37 modified flaps (2.7%) and in 6/11 retained flaps (54.5%) (Fisher’s exact test, *p* < 0.001; Firth OR, 0.035; 95% CI, 0.003–0.208) (Table 3).

### 3.4. Firth Penalized Logistic Regression

Univariable Firth penalized associations of the candidate variables with overall and severe flap necrosis are presented in Table S3. For overall flap necrosis, Group B was associated with lower odds than Group A in the primary model adjusted for transfer method (OR, 0.223; 95% CI, 0.063–0.661; *p* = 0.006) and in the extended model additionally adjusted for defect area (OR, 0.222; 95% CI, 0.063–0.657; *p* = 0.006). For severe flap necrosis, Group B was also associated with lower odds in the primary adjusted model (OR, 0.097; 95% CI, 0.010–0.436; *p* = 0.001) and in the extended model (OR, 0.095; 95% CI, 0.010–0.437; *p* = 0.001) (Table 4).

### 3.5. Sensitivity Analyses

In a sensitivity analysis excluding the 11 flaps with ICGA-detected hypoperfusion that were retained without modification (*n* = 97), overall flap necrosis occurred in 4/59 flaps (6.8%) in Group B and in 8/38 flaps (21.1%) in Group A (Fisher’s exact test, *p* = 0.056; Firth OR, 0.291; 95% CI, 0.078–0.957). Severe flap necrosis occurred in 1/59 flaps (1.7%) and in 5/38 flaps (13.2%), respectively (Fisher’s exact test, *p* = 0.033; Firth OR, 0.156; 95% CI, 0.016–0.828).

When the cohort was restricted to procedures performed from 2016 onward (*n* = 80), overall flap necrosis occurred in 4/59 flaps (6.8%) in Group B and in 7/21 flaps (33.3%) in Group A (Fisher’s exact test, *p* = 0.006; Firth OR, 0.157; 95% CI, 0.039–0.561). Severe flap necrosis occurred in 1/59 flaps (1.7%) and in 7/21 flaps (33.3%), respectively (Fisher’s exact test, *p* < 0.001; Firth OR, 0.050; 95% CI, 0.005–0.253).

When both restrictions were applied simultaneously (*n* = 69), Group A comprised 10 flaps. Overall flap necrosis occurred in 4/59 flaps (6.8%) and in 1/10 flaps (10.0%) (Fisher’s exact test, *p* = 0.555; Firth OR, 0.514; 95% CI, 0.082–5.531). Severe flap necrosis occurred in 1/59 flaps (1.7%) and in 1/10 flaps (10.0%) (Fisher’s exact test, *p* = 0.271; Firth OR, 0.162; 95% CI, 0.012–2.155) (Table 5).

## 4. Discussion

### 4.1. Principal Findings and Interpretation

ICGA provides real-time visualization of flap perfusion during reconstructive surgery [3,4,7]. Reported associations between ICGA use and postoperative flap outcomes have varied across studies [7,8,9,10,11,12,13,14,15,16,17,18]. As summarized in Table S1, this variation may relate to differences in flap type and reconstructive setting, definitions of postoperative outcomes, and the extent to which ICGA findings guided final intraoperative decision-making. To address these sources of variation, this study examined a single flap type, used overall and severe flap necrosis as the outcomes, and classified flaps according to the basis for final intraoperative decision-making rather than ICGA use alone. In this retrospective cohort, Group B showed lower observed rates and lower adjusted odds of both overall and severe flap necrosis than Group A, and the association remained similar in direction and magnitude across the unadjusted, primary adjusted, and extended Firth penalized models. In contrast, the secondary comparison based solely on whether ICGA was performed showed no clear difference in either outcome.

The two comparisons differ mainly because 11 flaps were classified differently. In these flaps, ICGA showed distal hypoperfusion, but no feasible modification could remove the poorly perfused tissue while preserving enough tissue to cover the defect. The tissue therefore had to be retained, and the final decision relied on clinical assessment. These flaps remained in the ICGA-assessed cohort in the secondary comparison but were placed in Group A in the primary comparison. This explains why the two analyses addressed different clinical questions. Group B included flaps in which ICGA either confirmed adequate perfusion or guided a feasible modification. Group A included flaps managed mainly by clinical assessment, including three that were modified on clinical grounds. The primary comparison therefore asks whether ICGA findings contributed to a safe and workable operative plan, rather than whether ICGA was simply performed. Clinically, this is the more relevant question because the practical value of ICGA depends on whether its findings can support a feasible decision. However, perfusion status, the feasibility of modification, and the modification actually performed were also linked to group assignment. The observed association therefore reflects the management pathway as a whole and cannot be attributed to ICGA alone.

The sensitivity analyses were performed to examine two concerns about the primary comparison. The first was whether the observed difference was mainly driven by the 11 hypoperfused flaps that had to be retained. The second was whether the difference reflected the nonoverlapping early period before both management pathways were in use. First, after the 11 flaps were excluded, the crude difference in overall necrosis was no longer statistically significant, but the difference in severe necrosis remained significant. The Firth estimate for overall necrosis also remained in the same direction. This suggests that the 11 flaps contributed to the overall necrosis result, whereas the difference in severe necrosis remained after their exclusion. Second, the differences remained when the analysis was limited to procedures performed from 2016 onward, when both management pathways were in use. The persistence of the differences during this period argues against the nonoverlapping early years as the sole explanation, although other changes over time cannot be completely excluded. Third, applying both restrictions left only 10 flaps in Group A. This analysis was too small to provide a reliable additional conclusion. A separate post hoc exploratory analysis addressed a related question. Among the 48 flaps with hypoperfusion detected by ICGA, necrosis was less common when a feasible modification could be performed than when the tissue had to be retained. This finding is clinically consistent with the main interpretation that detecting hypoperfusion is most useful when the finding can be translated into a safe operative response. Because modification was not randomly assigned, this comparison cannot separate the effect of modification from the clinical factors that made modification possible.

### 4.2. Comparison with Previous Evidence

One source of heterogeneity is how comparison groups have been defined across studies. Kapadia et al. [8] compared procedures with and without ICGA but failed to mention whether fluorescence findings altered intraoperative management. Duggal et al. [12] used both clinical assessment and ICGA to guide debridement without identifying the basis for the final decision. Other studies instead distinguished interventions guided by clinical assessment from those guided by ICGA findings [2,27,28]. These approaches address different clinical questions because ICGA use alone leaves it unclear whether its findings influenced the final operative decision.

Flap type heterogeneity is another source of inconsistency in comparative ICGA studies. Hembd et al. [7] noted that heterogeneous flap types and reconstructive techniques could confound analyses of fat necrosis and accordingly restricted their cohort to single-pedicle deep inferior epigastric perforator flaps. Singaravelu et al. [10] reported different associations across reconstructive settings involving the breast, head and neck, lower extremity, and abdominal wall reconstruction, with inconsistent imaging protocols and predominantly subjective interpretation across studies. To limit variation related to flap type, the present study focused on pre-expanded e-LTMC flaps, in which distal perfusion remains an important intraoperative consideration [24,25]. In this setting, management of distal hypoperfusion requires a balance between excision of tissue at risk and preservation of adequate defect coverage. Focusing on a single flap type reduced this source of variation and allowed assessment of the association between the basis for final intraoperative decision-making and postoperative flap necrosis.

Outcome definition is a further source of inconsistency. Momeni and Sheckter [11] observed that definitions of fat necrosis varied by lesion size, timing of assessment, and mode of detection, making comparative analysis difficult. Singaravelu et al. [10] reported different pooled effects for total and partial flap loss in head and neck reconstruction, illustrating how outcome selection can influence interpretation. Broad postoperative or wound-related endpoints combining tissue necrosis, infection, seroma, hematoma, wound dehiscence, and reoperation have also been used [8,13,16]. Such composite endpoints combine complications with different mechanisms and therefore provide a less focused measure of perfusion-related flap necrosis.

In the present study, overall flap necrosis and severe flap necrosis were assessed as distinct outcomes. Severe necrosis was defined as clinically significant tissue loss requiring active wound management beyond routine postoperative care rather than according to the anatomical depth of tissue loss. This treatment-burden definition reflects the clinical consequences of necrosis more directly than broader composite endpoints. However, it may also be influenced by the quality of clinical documentation and local management practices. Prospective comparative studies should prespecify the flap population, the basis for final intraoperative decision-making, and outcome definitions before assessing whether the association extends across other flap types and reconstructive settings.

These considerations are also relevant when comparing the present study with our previous report [22]. The previous report described perfusion findings and their intraoperative management within an ICGA-assessed cohort. The present study expands that cohort to include flaps in which ICGA was not performed, allowing ICGA use to be distinguished from how ICGA findings informed the final decision. The present analysis also incorporates Firth penalized modeling, three sensitivity analyses, and a post hoc analysis of flaps with ICGA-detected hypoperfusion. Because 69 of the 108 flaps were included in the earlier report, the present study is an expanded exploratory analysis of a partially overlapping cohort rather than an independent validation. The two studies also used different definitions of necrosis severity. The treatment-burden classification used in the present study is not directly interchangeable with the anatomical partial-thickness and full-thickness classification used previously.

### 4.3. Clinical Implications

Although Group B showed lower observed rates of overall flap necrosis and severe flap necrosis, necrosis still occurred in four flaps, including one severe case. These cases illustrate that ICGA provides a time-specific assessment of perfusion rather than a guarantee of postoperative flap survival. In this study, the ICGA examination used for final decision-making was performed after flap transfer and inset. However, perfusion may still change afterward because of edema, external compression, or evolving microcirculatory failure. Flap rotation and passage through a subcutaneous tunnel may also alter perfusion between examinations [29]. Qualitative fluorescence interpretation may introduce variability in delineating hypoperfused boundaries because no predefined quantitative threshold was used. Quantitative ICGA assessment may improve objectivity. However, a broad intermediate range may remain in which tissue viability cannot be predicted with confidence [30]. Values within this range may not clearly guide whether the tissue should be retained, excised, or trimmed, although this is the decision the surgeon ultimately needs to make. Reproducibility is further affected by differences in equipment, working distance, ICG dose, and acquisition timing. These factors limit comparability across studies [31].

These findings have practical implications. ICGA should support rather than replace conventional clinical assessment. A hypoperfused area on ICGA should not automatically be excised. The surgeon must also consider the extent of hypoperfusion, the size of the defect, and the amount of tissue required for coverage. When a safe modification is possible, ICGA can help identify tissue at risk and guide prophylactic excision or distal trimming. When the hypoperfused tissue must be retained to maintain adequate coverage, closer postoperative monitoring becomes particularly important. Prospective studies should determine whether standardized imaging timing and reproducible quantitative assessment improve the consistency of ICGA-informed intraoperative decision-making.

### 4.4. Limitations

This study has several limitations. Its retrospective and nonrandomized design prevents causal interpretation. These analytical groups were constructed retrospectively for the present analysis rather than prospectively assigned at the time of surgery. Because flaps were classified according to the basis for the final intraoperative decision, group allocation was influenced by perfusion status, the feasibility of modification, coverage requirements, the modification actually performed, and surgeon judgment. Selection bias and confounding by indication therefore remain despite statistical adjustment, and the separate contributions of ICGA findings and flap modification cannot be determined. The same concern applies to the post hoc analysis of the 48 flaps with ICGA-detected distal hypoperfusion, particularly because only 11 flaps were retained (Table S2).

The two management pathways overlapped only from 2016 onward. Restricting the analysis to this period mitigated temporal confounding without eliminating it, and changes in surgical experience, case selection, and perioperative care remain possible. Outcome events were also sparse, with 18 overall and 12 severe necrosis events and only one severe event in Group B. Firth penalized logistic regression reduced small-sample and separation bias, but it could not overcome the limited amount of information or residual confounding.

ICGA interpretation was qualitative, and outcome assessment was retrospective and not blinded to the intraoperative management pathway. The treatment-burden definition of severity may also have been influenced by documentation quality and local management practice. Generalizability is limited by the single-center, single-surgeon design, the uncommon flap type, and the predominance of post-burn neck reconstruction. In addition, 69 of the 108 flaps overlapped with our previous report [22]. Nevertheless, the present study provides a clinically detailed evaluation of how ICGA findings were incorporated into intraoperative decisions within a single flap type and offers a basis for prospective validation.

## 5. Conclusions

In this retrospective exploratory cohort of pre-expanded e-LTMC flaps, a management pathway in which ICGA findings informed or confirmed the final intraoperative decision was associated with lower observed rates and lower adjusted odds of overall and severe flap necrosis. Because perfusion status and the feasibility of modification also influenced the final decision, the findings reflect the management pathway as a whole rather than an isolated effect of ICGA. The association with overall necrosis was attenuated after the retained hypoperfused flaps were excluded, and the most restrictive analysis was imprecise. ICGA should therefore be used as an adjunct to clinical assessment, and prospective multicenter studies with contemporaneous comparison groups, prespecified management algorithms, standardized interpretation, and independent outcome assessment are needed.

## Figures and Tables

**Table 1:** Baseline and Operative Characteristics According to the Basis for the Final Intraoperative Decision.

Characteristic	Overall (*n* = 108)	Group A (*n* = 49)	Group B (*n* = 59)	*p* Value
Age, years	16.0 ± 11.0	16.4 ± 12.5	15.6 ± 9.8	0.703
Male sex	61 (56.5)	26 (53.1)	35 (59.3)	0.562
Body mass index, kg/m^2^	19.8 ± 3.6	20.1 ± 3.9	19.4 ± 3.4	0.346
Overweight or obesity	26 (24.1)	13 (26.5)	13 (22.0)	0.654
Smoking history	5 (4.6)	1 (2.0)	4 (6.8)	0.374
Etiology: post-burn scar contracture	98 (90.7)	43 (87.8)	55 (93.2)	0.507
Defect location: neck	96 (88.9)	41 (83.7)	55 (93.2)	0.135
Defect length, cm	24.7 ± 5.8	25.4 ± 6.7	24.2 ± 5.0	0.264
Defect width, cm	11.8 ± 2.4	11.0 ± 2.5	12.4 ± 2.2	0.003
Defect area, cm^2^	297 ± 105	289 ± 124	303 ± 85	0.506
Expander volume, mL	762 ± 304	800 ± 363	731 ± 243	0.256
Expansion duration, weeks	27.5 ± 8.7	24.8 ± 6.5	29.8 ± 9.7	0.002
Flap length, cm	35.6 ± 6.9	35.2 ± 7.9	36.0 ± 6.0	0.583
Flap width, cm	12.8 ± 2.1	12.5 ± 2.2	13.0 ± 2.1	0.221
Flap area, cm^2^	462 ± 140	449 ± 155	472 ± 126	0.401
Relative flap length, ratio	1.21 ± 0.12	1.18 ± 0.11	1.23 ± 0.12	0.026
DSA–Tuffier’s line distance, cm	29.6 ± 5.6	29.9 ± 6.4	29.4 ± 5.0	0.677
Flap extending beyond Tuffier’s line	90 (83.3)	38 (77.6)	52 (88.1)	0.195
Unexpanded distal flap component	42 (38.9)	11 (22.4)	31 (52.5)	0.002
Transfer through a subcutaneous tunnel	84 (77.8)	34 (69.4)	50 (84.7)	0.066

Values are presented as mean ± standard deviation or *n* (%). *p* values were calculated using Welch’s *t* test for continuous variables and Fisher’s exact test for categorical variables. Overweight or obesity was defined using the age- and sex-specific International Obesity Task Force body mass index cutoffs. Relative flap length was calculated as flap length divided by the DSA–Tuffier’s line distance. An unexpanded distal flap component was recorded when present on intraoperative inspection. Group A denotes the clinical-assessment group, and Group B denotes the ICGA-guided group. DSA, dorsal scapular artery; ICGA, indocyanine green angiography.

**Table 2:** Intraoperative Decision Pathways and Postoperative Flap Necrosis Outcomes of the 108 Flaps.

Pathway	Group	*n*	Necrosis Status, *n* (%)			
			None	Mild	Severe	Overall
1. Clinical assessment alone; no ICGA	A	38	30 (78.9)	3 (7.9)	5 (13.2)	8 (21.1)
2. Hypoperfusion; retained	A	11	5 (45.5)	0 (0.0)	6 (54.5)	6 (54.5)
3. Adequate perfusion	B	22	21 (95.5)	1 (4.5)	0 (0.0)	1 (4.5)
4. Prophylactic excision	B	32	29 (90.6)	2 (6.2)	1 (3.1)	3 (9.4)
5. Distal trimming to FTSG	B	5	5 (100.0)	0 (0.0)	0 (0.0)	0 (0.0)
Total	—	108	90 (83.3)	6 (5.6)	12 (11.1)	18 (16.7)
Group A	—	49	35 (71.4)	3 (6.1)	11 (22.4)	14 (28.6)
Group B	—	59	55 (93.2)	3 (5.1)	1 (1.7)	4 (6.8)
ICGA performed	—	70	60 (85.7)	3 (4.3)	7 (10.0)	10 (14.3)
ICGA not performed	—	38	30 (78.9)	3 (7.9)	5 (13.2)	8 (21.1)

Percentages are calculated within each row. Overall necrosis includes mild and severe necrosis. Mild necrosis resolved with routine postoperative care or conservative dressing changes; severe necrosis required active wound management beyond routine postoperative care. Group A comprises pathways 1 and 2, and Group B comprises pathways 3–5. Pathways 2, 4, and 5 comprised flaps with ICGA-detected distal hypoperfusion. The three flaps modified without ICGA remained in pathway 1 because management was based on clinical assessment. FTSG, full-thickness skin graft; ICGA, indocyanine green angiography.

**Table 3:** Unadjusted Comparisons of Postoperative Flap Necrosis.

Comparison	Outcome	Index *n*/*N* (%)	Reference *n*/*N* (%)	Fisher *p*	Firth OR (95% CI)
C1. Group B vs. Group A	Overall necrosis	4/59 (6.8)	14/49 (28.6)	0.004	0.199 (0.057–0.581)
	Severe necrosis	1/59 (1.7)	11/49 (22.4)	0.001	0.086 (0.009–0.383)
C2. ICGA vs. no ICGA	Overall necrosis	10/70 (14.3)	8/38 (21.1)	0.422	0.623 (0.228–1.737)
	Severe necrosis	7/70 (10.0)	5/38 (13.2)	0.750	0.719 (0.222–2.456)
C3. Modified vs. retained	Overall necrosis	3/37 (8.1)	6/11 (54.5)	0.002	0.086 (0.016–0.393)
	Severe necrosis	1/37 (2.7)	6/11 (54.5)	<0.001	0.035 (0.003–0.208)

C1 compares Group B with Group A; C2 compares flaps with and without ICGA; and C3 compares modified with retained flaps among the 48 flaps with ICGA-detected distal hypoperfusion. C1 was the primary comparison, C2 was the secondary comparison, and C3 was a post hoc exploratory comparison. Odds ratios and 95% confidence intervals were estimated using univariable Firth penalized logistic regression; *p* values were calculated using Fisher’s exact test. CI, confidence interval; ICGA, indocyanine green angiography; OR, odds ratio.

**Table 4:** Association Between the Basis for the Final Intraoperative Decision and Postoperative Flap Necrosis in Firth Penalized Logistic Regression Models.

Outcome	Model	Events/EPV	OR (95% CI)	*p* Value
Overall flap necrosis	Unadjusted	18/18.0	0.199 (0.057–0.581)	0.003
	Adjusted for transfer method	18/9.0	0.223 (0.063–0.661)	0.006
	Adjusted for transfer method and defect area	18/6.0	0.222 (0.063–0.657)	0.006
Severe flap necrosis	Unadjusted	12/12.0	0.086 (0.009–0.383)	0.001
	Adjusted for transfer method	12/6.0	0.097 (0.010–0.436)	0.001
	Adjusted for transfer method and defect area	12/4.0	0.095 (0.010–0.437)	0.001

Odds ratios compare Group B with Group A, with Group A as the reference. Confidence intervals are profile penalized-likelihood intervals, and *p* values are from penalized likelihood-ratio tests. EPV was calculated as the number of outcome events divided by the number of model parameters excluding the intercept. CI, confidence interval; EPV, events per variable; OR, odds ratio.

**Table 5:** Sensitivity Analyses of the Primary Comparison Between the ICGA-Guided Group and the Clinical-Assessment Group.

Analysis	Outcome	Group B *n*/*N* (%)	Group A *n*/*N* (%)	Fisher *p*	Firth OR (95% CI)
S1. Exclude retained hypoperfusion (*n* = 97)	Overall necrosis	4/59 (6.8)	8/38 (21.1)	0.056	0.291 (0.078–0.957)
	Severe necrosis	1/59 (1.7)	5/38 (13.2)	0.033	0.156 (0.016–0.828)
S2. From 2016 onward (*n* = 80)	Overall necrosis	4/59 (6.8)	7/21 (33.3)	0.006	0.157 (0.039–0.561)
	Severe necrosis	1/59 (1.7)	7/21 (33.3)	<0.001	0.050 (0.005–0.253)
S3. Both restrictions (*n* = 69)	Overall necrosis	4/59 (6.8)	1/10 (10.0)	0.555	0.514 (0.082–5.531)
	Severe necrosis	1/59 (1.7)	1/10 (10.0)	0.271	0.162 (0.012–2.155)

S1 excluded flaps with ICGA-detected distal hypoperfusion that were retained without modification; S2 included procedures performed from 2016 onward; and S3 applied both restrictions. *p* values were calculated using Fisher’s exact test. Odds ratios and 95% confidence intervals were estimated using univariable Firth penalized logistic regression, with Group A as the reference. CI, confidence interval; ICGA, indocyanine green angiography; OR, odds ratio.

## Data Availability

The data presented in this study are available on reasonable request from the corresponding author. The data are not publicly available due to patient privacy restrictions.

## Supplementary Materials

The following supporting information can be downloaded at: https://www.mdpi.com/article/10.3390/jcm15166262/s1, Table S1: Published Comparative Studies of Intraoperative ICGA; Table S2: Baseline and Operative Characteristics of the 48 Flaps with ICGA-Detected Distal Hypoperfusion; Table S3: Univariable Firth Penalized Associations of Candidate Variables with Overall and Severe Flap Necrosis; Video S1: First 136 s segment of a continuous ICGA fluorescence recording; Video S2: Subsequent 136 s segment of the same continuous ICGA fluorescence recording.
