# Supplementary material for: ICGA-Guided Decision-Making Is Associated with Lower Flap Necrosis Rates in Pre-Expanded Extended Lower Trapezius Myocutaneous Flaps
Source: J Clin Med. 2026 Aug 13;15(16):6262. doi: 10.3390/jcm15166262 (PMC13513236; doi:10.3390/jcm15166262)
Supplement: Supplementary file 1 [file jcm-15-06262-s001.zip › jcm-4453000-supplemrntary tables.pdf]

Table S1. Published Comparative Studies of Intraoperative ICGA

Year	Author	Study design	Sample size (ICGA vs control)	Applications studied	Flap modification	Primary outcome	Result
2022	Yoo	Retrospective review	187 vs 110	Microsurgical breast reconstruction	Yes	Fat necrosis	(Negative) No statistically significant difference in incidence of postoperative fat necrosis was found.
2021	Kapadia	Retrospective review	36 vs 40	Lower extremity reconstruction	Not stated	Postoperative complications	(Negative) The total number of postoperative complications did not statistically differ between cohorts.
2020	Varela	Randomized controlled trial	24 vs 27	Microsurgical breast reconstruction	Yes	Fat necrosis	(Positive) ICGA significantly reduced the incidence of fat necrosis.
2020	Shao	Retrospective review	88 vs 58	Abdominal wall reconstruction	Yes	Surgical site occurrence	(Negative) There was no statistical difference in incidence of surgical site occurrence.
2020	Momeni	Retrospective review	58 vs 79	Microsurgical breast reconstruction	Yes	Fat necrosis	(Positive) Intraoperative use of ICGA was associated with significantly lower odds of fat necrosis.
2020	Hembd	Retrospective review	200 vs 306	Microsurgical breast reconstruction	Yes	Fat necrosis	(Positive) Intraoperative ICGA decreases the odds of fat necrosis.
2019	Malagon-Lopez	Prospective study	24 vs 37	Microsurgical breast reconstruction	Yes	Fat necrosis	(Positive) ICGA reduce the incidence of fat necrosis by half as well as reduce its grade.
2018	Mirhaidari	Prospective study	126 vs 117	Postmastectomy skin flap	Yes	Flap necrosis	(Negative) The rate of flap necrosis in the ICGA group was lower than the historical group with no statistical significance.
2018	Hammer-Hansen	Retrospective review	66 vs 62	Implant-based breast reconstruction	Yes	Flap necrosis	(Negative) No significant difference in necrosis rates were found.
2018	Alstrup	Retrospective review	77 vs 114	Autologous breast reconstructions	Yes	Postoperative complications	(Negative) There was no significant difference in overall complication rates between the two groups.
2017	Gorai	Retrospective review	81 vs 100	Postmastectomy skin flap	Yes	Flap necrosis	(Positive) ICGA-guided trimming decreased the rate of deep skin necrosis requiring additional surgical treatment.
2016	Wormer	Randomized controlled trial	46 vs 49	Abdominal wall reconstruction	Yes	Wound complications	(Negative) ICGA guidance and intraoperative modification of flaps did not prevent wound-related complications or reoperation.
2016	Harless	Retrospective review	213 vs 254	Implant-based breast reconstruction	Yes	Flap necrosis	(Positive) The incidence of mastectomy flap skin necrosis prior to ICGA was significantly higher.
2016	Diep	Retrospective review	61 vs 53	Postmastectomy skin flap	Yes	Flap necrosis	(Negative) The overall rates of flap necrosis were not significantly different.
2015	Casey	Retrospective review	6 vs 7	Microsurgical breast reconstruction	Yes	Flap loss and fat necrosis	(Positive) There was a statistically significant decrease in the rate of partial flap loss and fat necrosis.
2015	Beckler	Retrospective review	25 vs 48	Fibula flap	Yes	Flap necrosis	(Positive) ICGA may reduce the risk of partial skin flap necrosis.
2014	Duggal	Retrospective review	184 vs 184	Postmastectomy breast reconstruction	Not stated	Flap necrosis	(Positive) Patients who underwent procedures using ICGA had a lower incidence of mastectomy skin necrosis and unexpected reoperations for perfusion-related complications.
2013	Sood	Retrospective review	39 vs 52	Postmastectomy breast reconstruction	Yes	Postoperative complications	(Negative) The rate of postoperative complications was higher in the Pre-ICGA group compared to the Post- ICGA group, while this difference was not significant.
2013	Patel	Retrospective review	5 vs 12	Abdominal wall reconstruction	Yes	Wound-healing complications	(Positive) Intraoperative ICGA can decrease wound healing related complications.
2010	Komorowska-Timek	Retrospective review	24 vs 206	Microsurgical breast reconstruction	Yes	Postoperative complications	(Positive) The complication rate was significantly less than that observed in the previous patients.

Studies are listed in reverse chronological order. Sample sizes are given as the number in the ICGA group versus the number in the control group. Flap modification not stated indicates that the original report did not specify whether flaps were modified in response to the angiographic findings. (Positive) indicates that the cited study reported a statistically significant difference favoring ICGA, and (Negative) indicates that it did not. Findings are reported as concluded by the original authors and were not re-analyzed for the present study. ICGA, indocyanine green angiography.

Table S2. Baseline and Operative Characteristics of the 48 Flaps With ICGA-Detected Distal Hypoperfusion

Characteristic	Retained (n = 11)	Modified (n = 37)	*P* value
Age, years	17.2 ± 11.7	16.1 ± 10.4	0.788
Body mass index, kg/m²	21.3 ± 2.8	19.5 ± 2.9	0.076
Defect area, cm²	352 ± 168	302 ± 78	0.363
Flap area, cm²	508 ± 191	472 ± 110	0.560
Flap length, cm	37.4 ± 7.4	36.2 ± 5.0	0.646
Relative flap length, ratio	1.21 ± 0.10	1.27 ± 0.11	0.144
Transfer through a subcutaneous tunnel	8 (72.7)	32 (86.5)	0.361
Flap extending beyond Tuffier's line	10 (90.9)	35 (94.6)	0.551
Unexpanded distal flap component	6 (54.5)	24 (64.9)	0.724

Values are presented as mean ± standard deviation or n (%). P values were calculated using Welch’s t test for continuous variables and Fisher’s exact test for categorical variables. Retained denotes flaps retained without modification; modified denotes flaps treated by prophylactic excision or distal trimming for use as a full-thickness skin graft. FTSG, full-thickness skin graft; ICGA, indocyanine green angiography.

Table S3. Univariable Firth Penalized Associations of Candidate Variables With Overall and Severe Flap Necrosis

Variable	Overall flap necrosis		Severe flap necrosis	
	OR (95% CI)	*P* value	OR (95% CI)	*P* value
Group B vs Group A	0.199 (0.057–0.581)	0.003	0.086 (0.009–0.383)	0.001
ICGA performed	0.623 (0.228–1.737)	0.358	0.719 (0.222–2.456)	0.586
Age, per year	1.004 (0.958–1.048)	0.868	1.015 (0.961–1.065)	0.581
Male sex	2.151 (0.767–6.811)	0.149	1.063 (0.331–3.613)	0.918
Body mass index, per unit	0.963 (0.830–1.104)	0.597	1.086 (0.926–1.270)	0.301
Overweight or obesity	1.317 (0.407–3.830)	0.629	2.575 (0.740–8.538)	0.132
Smoking history	1.648 (0.158–9.651)	0.623	2.681 (0.253–16.286)	0.357
Post-burn etiology	0.680 (0.167–3.841)	0.625	0.403 (0.094–2.345)	0.280
Neck lesion	0.861 (0.221–4.774)	0.844	1.031 (0.211–10.122)	0.973
Defect length, per cm	1.009 (0.924–1.096)	0.828	1.050 (0.951–1.152)	0.321
Defect width, per cm	0.961 (0.783–1.182)	0.703	1.138 (0.890–1.484)	0.308
Defect area, per cm^2	1.000 (0.995–1.005)	0.849	1.004 (0.999–1.009)	0.132
Expander volume, per mL	1.001 (1.000–1.003)	0.129	1.001 (1.000–1.003)	0.117
Expansion duration, per week	0.961 (0.893–1.022)	0.223	0.997 (0.922–1.060)	0.922
Flap length, per cm	0.997 (0.927–1.072)	0.930	1.028 (0.944–1.122)	0.531
Flap width, per cm	0.933 (0.738–1.178)	0.557	1.075 (0.817–1.438)	0.611
Flap area, per cm^2	1.000 (0.996–1.003)	0.798	1.002 (0.998–1.006)	0.409
Relative flap length, per unit	36.922 (0.442–4431.712)	0.112	4.239 (0.028–894.874)	0.582
DSA–Tuffier's line distance, per cm	0.966 (0.880–1.056)	0.453	1.015 (0.913–1.127)	0.784
Flap extending beyond Tuffier's line	2.778 (0.626–26.316)	0.199	1.688 (0.362–16.274)	0.543
Unexpanded distal flap component	0.581 (0.183–1.635)	0.311	0.804 (0.219–2.617)	0.723
Subcutaneous tunnel vs propeller	0.365 (0.127–1.082)	0.068	0.343 (0.103–1.201)	0.092
Surgical year, per year	1.022 (0.824–1.249)	0.839	1.021 (0.791–1.247)	0.871

Each row represents a separate univariable Firth penalized logistic regression model. Confidence intervals are profile penalized-likelihood intervals, and P values are from penalized likelihood-ratio tests. These analyses were not used to select covariates for the multivariable models. Variable definitions are provided in Table 1. CI, confidence interval; DSA, dorsal scapular artery; ICGA, indocyanine green angiography; OR, odds ratio.
